# Supplementary material for: Evidence of Immune Modulators in the Secretome of the Equine Tapeworm Anoplocephala perfoliata
Source: Pathogens. 2021 Jul 20;10(7):912. doi: 10.3390/pathogens10070912 (PMC8308605; doi:10.3390/pathogens10070912)
Supplement: Supplementary file 1 [file Supplementary Figure S4. A. perfoliata alpha Enolase multiple sequences alignment and secondary structure prediction.pdf › Supplementary Figure S4. A. perfoliata alpha Enolase multiple sequences alignment and secondary structure prediction.pdf]

```
                             10         20         30         40         50         60         70         80         90        100
Apα-Enolase-1                ---------- ---------- ---------- ---------- ---------- ---------- ---------- ---------- ---------- ----------
Apα-Enolase-2                ---------- ---------- ---------- ---------- ---------- ---------- ---------- ---------- ---------- ----------
Apα-Enolase-3                ---------- ---------- ---------- ---------- ---------- ---------- ---------- ---------- ---------- ----------
AAP81756_Ovol_Enolase        ---------- ---------- ---------- ---------- ---------- ---------- ---------- ---------- ---------- ----------
THD20548_Fhep_Enolase        ---------- ---------- ---------- ---------- ---------- ---------- ---------- ---------- ---------- ----------
KAG5442501_Csin_Enolase      ---------- ---------- ---------- ---------- ---------- ---------- ---------- ---------- ---------- ----------
Q27877_Sman_Enolase          ---------- ---------- ---------- ---------- ---------- ---------- ---------- ---------- ---------- ----------
XP_012793992_Shae_Enolase    MAYLRTGAEF ISSYRSSLNR MSELQKKIKM FGFNNAYYNQ VGLLKHDWLP HSPVWREALR RLPRELQEAR DFRISRATHL YASKNILPKE EWITIEDDIP
CDS37852_Emul_Enolase        ---------- ---------- ---------- ---------- ---------- ---------- ---------- ---------- ---------- ----------
ACY30465_Egran_Enolase       ---------- ---------- ---------- ---------- ---------- ---------- ---------- ---------- ---------- ----------
CDS30005_Hmic_Enolase        ---------- ---------- ---------- ---------- ---------- ---------- ---------- ---------- ---------- ----------
CDS26422_Hmic_Enolase        ---------- ---------- ---------- ---------- ---------- ---------- ---------- ---------- ---------- ----------
CAA59331_Hsap_Enolase        ---------- ---------- ---------- ---------- ---------- ---------- ---------- ---------- ---------- ----------
NP_001020559_Mmus_Enolase    ---------- ---------- ---------- ---------- ---------- ---------- ---------- ---------- ---------- ----------
ADQ00605_Asum_Enolase        ---------- ---------- ---------- ---------- ---------- ---------- ---------- ---------- ---------- ----------
AIZ75644_Hcon_Enolase        ---------- ---------- ---------- ---------- ---------- ---------- ---------- ---------- ---------- ----------
AHB59732_Tsol_Enolase        ---------- ---------- ---------- ---------- ---------- ---------- ---------- ---------- ---------- ----------

                            110        120        130        140        150        160        170        180        190        200
                                                                                            Enolase N
Apα-Enolase-1                ---------- ---------- ---------- -------MSI SKIHARQIFD SRGNPTVEVD LYTAKGMFRA AVPSGASTGI HEAVELRDGD KNAYMGKGVL
Apα-Enolase-2                ---------- ---------- ---------- --------M KSIVARQIFD SRGNPTVEVD LSTNKGCFRA AVPSGASTGI HEALELRDGI KTAYRGLGVQ
Apα-Enolase-3                ---------- ---------- ---------- -------MSI LKVNARQILD SRGNPTVEVD VITYKGLFRA SVPSGASAGI HEACELRDNN PQEFNGMGVL
AAP81756_Ovol_Enolase        ---------- ---------- ---------- -------MPI TRVHARPIYD SRGNPTVEVD LTTEKGIFRA AVPSGASTGI HEALELRDND EAVNHGKGVL
THD20548_Fhep_Enolase        ---------- ---------- ---------- -------MAI KAIHARQIFD SRGNPTVEVD VTTAKGLFRA AVPSGASTGV HEALELRD-G PPGYMGKGVL
KAG5442501_Csin_Enolase      ---------- ---------- ---------- -------MAI KAIHARQIFD SRGNPTVEVD VTTDKGLFRA AVPSGASTGV HEALELRD-G GKVYMGKGVM
Q27877_Sman_Enolase          ---------- ---------- ---------- -------MSI LTIHARQIFD SRGNPTVEVD LKTSKGLFRA AVPSGASTGV HEALELRDTN SKAYMKKGVL
XP_012793992_Shae_Enolase    YLDPYVEIVV KELNDRSNWD NFVNPEIYTN HNFSPQKMSI ISIHARQIFD SRGNPTVEVD LKTSKGLFRA AVPSGASTGV HEALELRDTK SKAYMGKGVL
CDS37852_Emul_Enolase        ---------- ---------- ---------- -------MSI LKIHARQIFD SRGNPTVEVD LTTSKGLFRA AVPSGASTGV HEAVELRDAD KNAYMGKGVL
ACY30465_Egran_Enolase       ---------- ---------- ---------- -------MSI LKIHARQIFD SRGNPTVEVD LTTSKGLFRA AVPSGASTGV HEAVELRDAD KNAYMGKGVL
CDS30005_Hmic_Enolase        ---------- ---------- ----MYSLLL SAGIALKMSI LKIHARQIFD SRGNPTVEVD LFTDHGMFRA AVPSGASTGI HEAVELRDND KANYMGKGVL
CDS26422_Hmic_Enolase        ---------- ---------- ---------- -------MSI LKITARQILD SRGNPTVEVD VITHKGLFRA SVPSGSSKGI HEAFELRDNN PLEFSGKGVR
CAA59331_Hsap_Enolase        ---------- ---------- ---------- -------MSI LKIHAREIFD SRGNPTVEVD LFTSKGLFRA AVPSGASTGI YEALELRDND KTRYMGKGVS
NP_001020559_Mmus_Enolase    ---------- ---------- ---------- -------MSI LRIHAREIFD SRGNPTVEVD LYTAKGLFRA AVPSGASTGI YEALELRDND KTRFMGKGVS
ADQ00605_Asum_Enolase        ---------- ---------- ---------- -------MPI TRIHARQIYD SRGNPTVEVD LTTEKGIFRA AVPSGASTGV HEALELRDKD KAVHHGKGVL
AIZ75644_Hcon_Enolase        ---------- ---------- ---------- -------MPI TKIHARQIYD SRGNPTVEVD LYTEKGVFRA AVPSGASTGV HEALELRDQD KKVHHGKGVL
AHB59732_Tsol_Enolase        ---------- ---------- ---------- -------MSI QKIHARQIFD SRGNPTVEVD LTTAKGMFRA AVPSGASTGV HEAVELRDGD KNAYMGKGVL
                                                                         β1              β2         β3                             α1

                            210        220        230        240        250        260        270        280        290        300
                                                  Enolase N                                                       Enolase C
Apα-Enolase-1                HAVKNVNDII APALIKEGLK VTEQEKIDNF MI-KLDGTKN KGKLGANAIL GVSLAACKAG AAEKGVPLYR YIASLAGNKD VILPVPAFNV LNGGSHAGNK
Apα-Enolase-2                KAVDNVNRII GPALCQRNFN MTNQEQIDMF MVKELDGSEN KSKLGANAIL GVSLAVAKAG AAEKNVPLYR HFADLAGNNE VILPVPSFNV LNGGSHAGNK
Apα-Enolase-3                DAVRNVKEKI GPALIKKDFH VTQQKEIDSF MVDELDGTPN KSRLGANAIL GVSLAVLKAG AAEKSIPLYR YVANMAGVTR VSLPVPAFNV INGGKHAGNN
AAP81756_Ovol_Enolase        QAVGNVNEQI GPALVAKNFC PTQQREIDLF ML-QLDGTEN KAKLGANAIL GVSLAVCKAG AVHKGMPLYK YIAELAGTRQ IVLPVPAMNV INGGSHAGNK
THD20548_Fhep_Enolase        KAVANVNSQI APNLIKSGIN VTDQAAVDKF ML-DLDGTPN KEKLGANAIL GVSLAVCKAG AAEKGLPLYK YIATLAGNKE VIMPVPSFNV INGGSHAGNK
KAG5442501_Csin_Enolase      KAVENVNKII APALLKCGIS VENQAEIDKI ML-DLDGTAN KEKLGANAIL GVSLAVCKAG AAQRNLPLYR HIASLAGNND VILPVPAFNV INGGSHAGNK
Q27877_Sman_Enolase          TAVSNVNKII APALINKNIP VTNQAAIDKY MI-DLDGTEN KEKLGANAIL GVSLAVCKAG AAEAGLPLYR YIARLAGHED VIMPVPAFNV INGGSHAGNK
XP_012793992_Shae_Enolase    TAVSNVNTTI APALIQKNIP VTDQAAIDRF MI-DLDGTEN KEKLGANAIL GVSLAVCKAG AAEAGLPLYR YIAKLAGHEN VIMPVPAFNV INGGSHAGNK
CDS37852_Emul_Enolase        NAVKNVNEII APALIKEKFL VTDQQRIDEF MI-KLDGSPN KGKLGANAIL GVSLAVCKAG AAEKGAPLYR HVADLAGNKD VVLPVPSFNV LNGGSHAGNK
ACY30465_Egran_Enolase       NAVKNVNEVI APALIKEKFV VTDQQRIDEF MI-KLDGSPN KGKLGANAIL GVSLAVCKAG AAEKGVPLYR HVADLAGNKD VVLPVPSFNV LNGGSHAGNK
CDS30005_Hmic_Enolase        KAVKNVNDII APELIKKGLK VTEQEAIDKF MI-ELDGTKN KGKLGANAIL GVSLAVCKAG AAQKGVPLYR YVAELAGNKN VFLPVPAFNV LNGGSHAGNK
CDS26422_Hmic_Enolase        KAVKNVNDII GPALIRKNFI VTRQEEIDNF MVKELDGTPN KCRLGANAIL GVSLAVLKAG AAEKCVPLYR YVANMAGVKR VSLPIPAFNV INGGRHAGNI
CAA59331_Hsap_Enolase        KAVEHINKTI APALVSKKLN VTEQEKIDKL MI-EMDGTEN KSKFGANAIL GVSLAVCKAG AVEKGVPLYR HIADLAGNSE VILPVPAFNV INGGSHAGNK
NP_001020559_Mmus_Enolase    QAVEHINKTI APALVSKKVN VVEQEKIDKL MI-EMDGTEN KSKFGANAIL GVSLAVCKAG AVEKGVPLYR HIADLAGNPE VILPVPAFNV INGGSHAGNK
ADQ00605_Asum_Enolase        NAVANINEKI GPAIVAKNFC PTQQVEIDKF ML-ALDGTEN KSNLGANAIL GVSLAVAKAG AVHKGMPLYK YIAELAGISQ VVLPVPAFNV INGGSHAGNK
AIZ75644_Hcon_Enolase        KAVANINDKI APALIAKNFC VTQQRDIDQF ML-ALDGTEN KSNLGANAIL GVSLAVAKAG AVHKGMPLYK YIAELAGVSK VILPVPAFNV INGGSHAGNK
AHB59732_Tsol_Enolase        NAVKNVNEVI APALLKEKFI VTDQEKIDEF MI-RLDGSPN KGKLGANAIL GVSLAVCKAG AAEKGVPLYR HIADLAGNKD VVLPVPSFNV LNGGSHAGNK
                                α1       α2              α3                             α4                   α5              β4

                            310        320        330        340        350        360        370        380        390        400
                                                                    Enolase C
Apα-Enolase-1                LAMQEFMILP TGAKSFTEAM KMGSEVYHNL KAVIKSKYGL DACNVGDEGG FAPNIQDNME GLELLKTAID KAGYTGKVKI GMDVAASEFY QDG--KYNLD
Apα-Enolase-2                LAFQEFMIMP TGATSFREAM KMGSETYHAL RAVIKAKYGL DACNVGDEGG FAPGIQDNFE ALELLNEAID NAGYSDRIRI ATDVAASEFY RDG--LYDLD
Apα-Enolase-3                LPIQEFLIFP TGAKSFAEAL RMGTETYHHL RNIICRKYGI SASGVGDEGG FTPNVANVYE ALDLLVEAIS TAKYSGKILI GIDVAASEFY ENG--KYNLG
AAP81756_Ovol_Enolase        LAMQEFMIMP VGASSFSEAM RMGSEIYHYL KAEIEKRYGL DATAVGDEGG FAPNIQDNKE GLDLLNTAIA TAGYTGKVSI AMDCAASEYS KEADKLYDLK
THD20548_Fhep_Enolase        LAMQEFMIMP TGASSFTEAM KIGSEVYHNL RAVIKSKYGL DACNVGDEGG FAPSIQDNLE GLELLRTAID KAGYTGKVKI AMDCAASEFY KEG--KYDLD
KAG5442501_Csin_Enolase      LAMQEFMIMP TGATSFKEAM QMGSEVYHNL KSVIKAKYGL DACNVGDEGG FAPNIQDNME GLQLLNEAIA KAGYTGKVKI AMDSAASEFH KDG--KYDLD
Q27877_Sman_Enolase          LAMQEFMILP TGASSFTEAM QIGTEVYHNL KAVIKREYGL DACNVGDEGG FAPNIQDNMK GLQLLEEAIK IAGYTGKVEI GMDCAASEFH KNG--KYDLD
XP_012793992_Shae_Enolase    LAMQEFMILP TGASSFTEAM KIGSEVYHNL KAVIKREYGL DACNVGDEGG FAPNIQDNMK GLQLLEEAIK IAGYTGKVEI GMDCAASEFH KNG--KYDLD
CDS37852_Emul_Enolase        LAMQEFMILP TGAKSFTEAM KMGTEVYHHL KSVIKGKYGL DACNVGDEGG FAPNIQDNME GLELLKTAID KAGYTGKVKI GMDVAASEFY QDG--KYNLD
ACY30465_Egran_Enolase       LAMQEFMILP TGAKSFSEAM KMGTEVYHHL KSVIKGKYGL DACNVGDEGG FAPNIQDNME GLELLKTAID KAGYTGKVKI GMDVAASEFY QDG--NYNLD
CDS30005_Hmic_Enolase        LAMQEFMILP TGAKNFTEAM KMGSEVYHHL KSVIKEKYGL DACNVGDEGG FAPNIQDNME GLELLNTAIA KAGYTGKVKI GMDVAASEFY QNG--KYNLD
CDS26422_Hmic_Enolase        LPMQEFLIFP VGAKSFSEAL QMGAETFHKL KNIILRRYGM VACNVGDEGG FAPSVSSANE ALDLLMEAID AAKYRGKILI GMNVAASTFY ENK--NYQLN
CAA59331_Hsap_Enolase        LAMQEFMILP VGAANFREAM RIGAEVYHNL KNVIKEKYGK DATNVGDEGG FAPNILENKE GLELLKTAIG KAGYTDKVVI GMDVAASEFS RSG--KYDLD
NP_001020559_Mmus_Enolase    LAMQEFMILP VGASSFREAM RIGAEVYHNL KNVIKEKYGK DATNVGDEGG FAPNILENKE ALELLKTAIA KAGYTDQVVI GMDVAASEFY RSG--KYDLD
ADQ00605_Asum_Enolase        LAMQEFMILP VGAKSFAEAM RMGSEIYHHL KAEIKKRYGL DATAVGDEGG FAPNIQDNKE GLDLLNTAIA LAGYTGRVSI GMDVAASEFY KAEEKKYDLD
AIZ75644_Hcon_Enolase        LAMQEFMILP VGATSFHEAM RMGSEVYHHL KAEIKKRYGL DATAVGDEGG FAPNIQDNKE GLDLLKTAID LAGYTGKISI GMDVAASEFY KQG--KYDLD
AHB59732_Tsol_Enolase        LAMQEFMILP TGAKNFTEAM KMGTEVYHHL KSVIKGKYGL DACNVGDEGG FAPNIQDNME GLELLKTAID RAGYTGKVKI GMDVAASEFY QDG--KYNLD
                                 β5                  α6                                         α7                β6      α8
```

Supplementary Figure S4. Multiple sequence alignment of the 3 novel α-Enolase from *Anoplocephala perfoliata* (Apα-Enolase-1, 2 and 3). Secondary protein structure prediction using the PSIPRED Protein Analysis Workbench is presented below the alignment and comprises of 10 β-strands and 15 α-helices, highlighted in yellow and pink, respectively. The 5 essential residues necessary for proper catalytic function are highlighted in green (Pancholi, 2001). The predicted N- and C-terminal Enolase domain profiles are indicated by blue- and red-boxes, respectively.

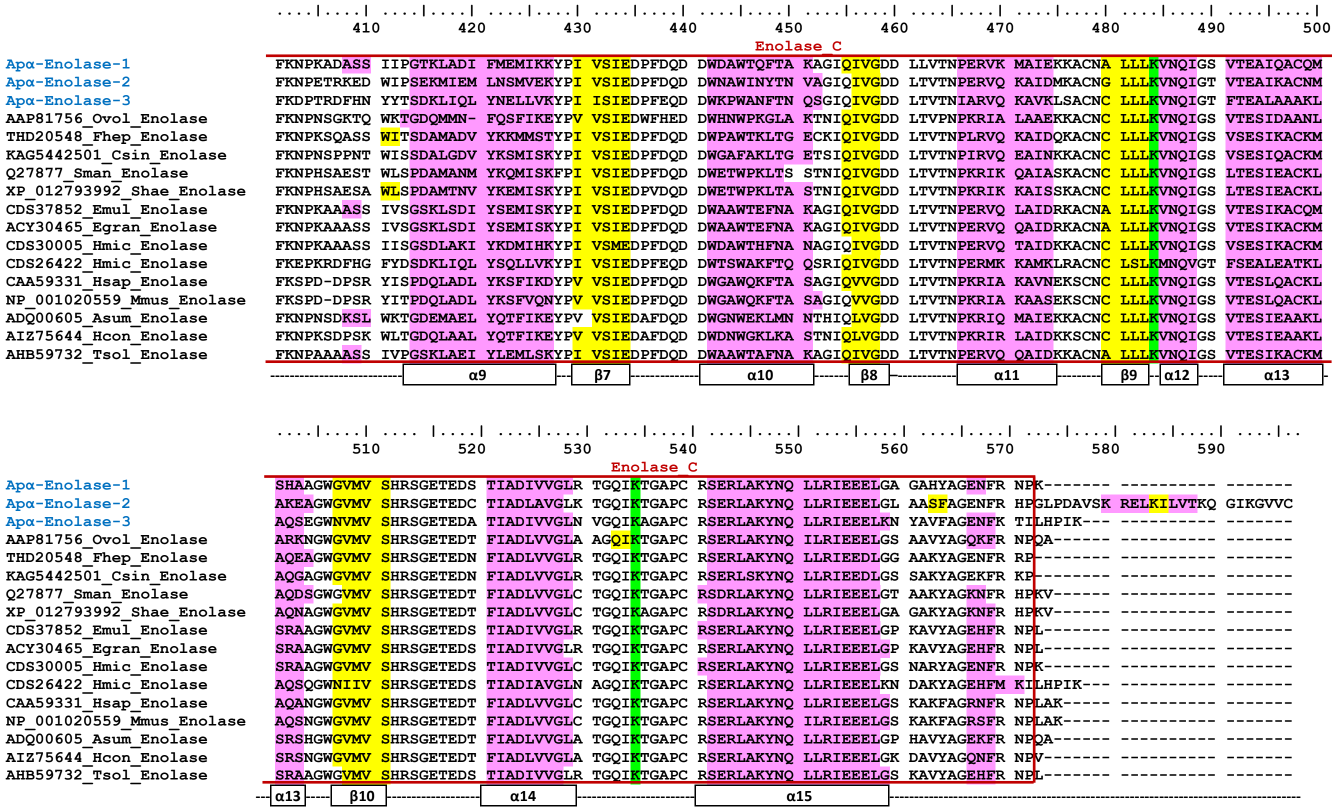

Supplementary Figure S4-Continued 2. Multiple sequence alignment of the 3 novel α-Enolase from *Anoplocephala perfoliata* (Apα-Enolase-1, 2 and 3). Secondary protein structure prediction using the PSIPRED Protein Analysis Workbench is presented below the alignment and comprises of 10 β-strands and 15 α-helices, highlighted in yellow and pink, respectively. The 5 essential residues necessary for proper catalytic function are highlighted in green (Pancholi, 2001). The predicted N- and C-terminal Enolase domain profiles are indicated by blue- and red-boxes, respectively.
