# Supplementary material for: Evidence of Immune Modulators in the Secretome of the Equine Tapeworm Anoplocephala perfoliata
Source: Pathogens. 2021 Jul 20;10(7):912. doi: 10.3390/pathogens10070912 (PMC8308605; doi:10.3390/pathogens10070912)
Supplement: Supplementary file 1 [file pathogens-10-00912-s001.zip › Supplementary Figure S1. Summary of the distribution of GO terms in the novel adult A. perfoliata transcriptome at level 3.pdf]

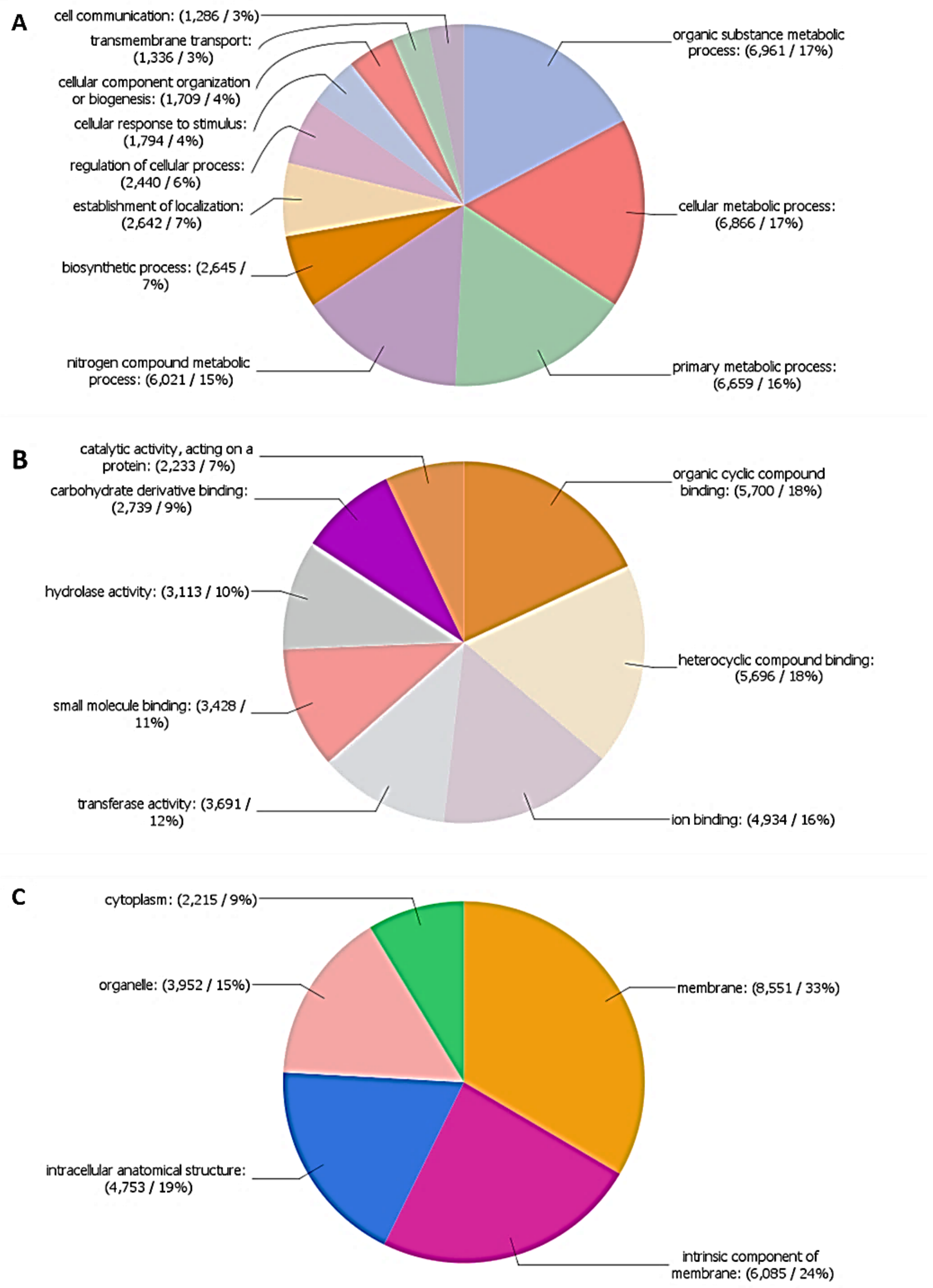

Supplementary Figure S1. Summary of the distribution of GO terms in the novel adult *Anoplocephala perfoliata* transcriptome at level 3 representing the relative abundance of GO terms in each three main categories. (A) Biological process category, (B) Molecular Functions category and (C) Cellular Components category
