# Supplementary material for: Evidence of Immune Modulators in the Secretome of the Equine Tapeworm Anoplocephala perfoliata
Source: Pathogens. 2021 Jul 20;10(7):912. doi: 10.3390/pathogens10070912 (PMC8308605; doi:10.3390/pathogens10070912)
Supplement: Supplementary file 1 [file pathogens-10-00912-s001.zip › Supplementary Figure S2. A. perfoliata Sigma class GST multiple sequences alignment and secondary structure prediction.pdf]

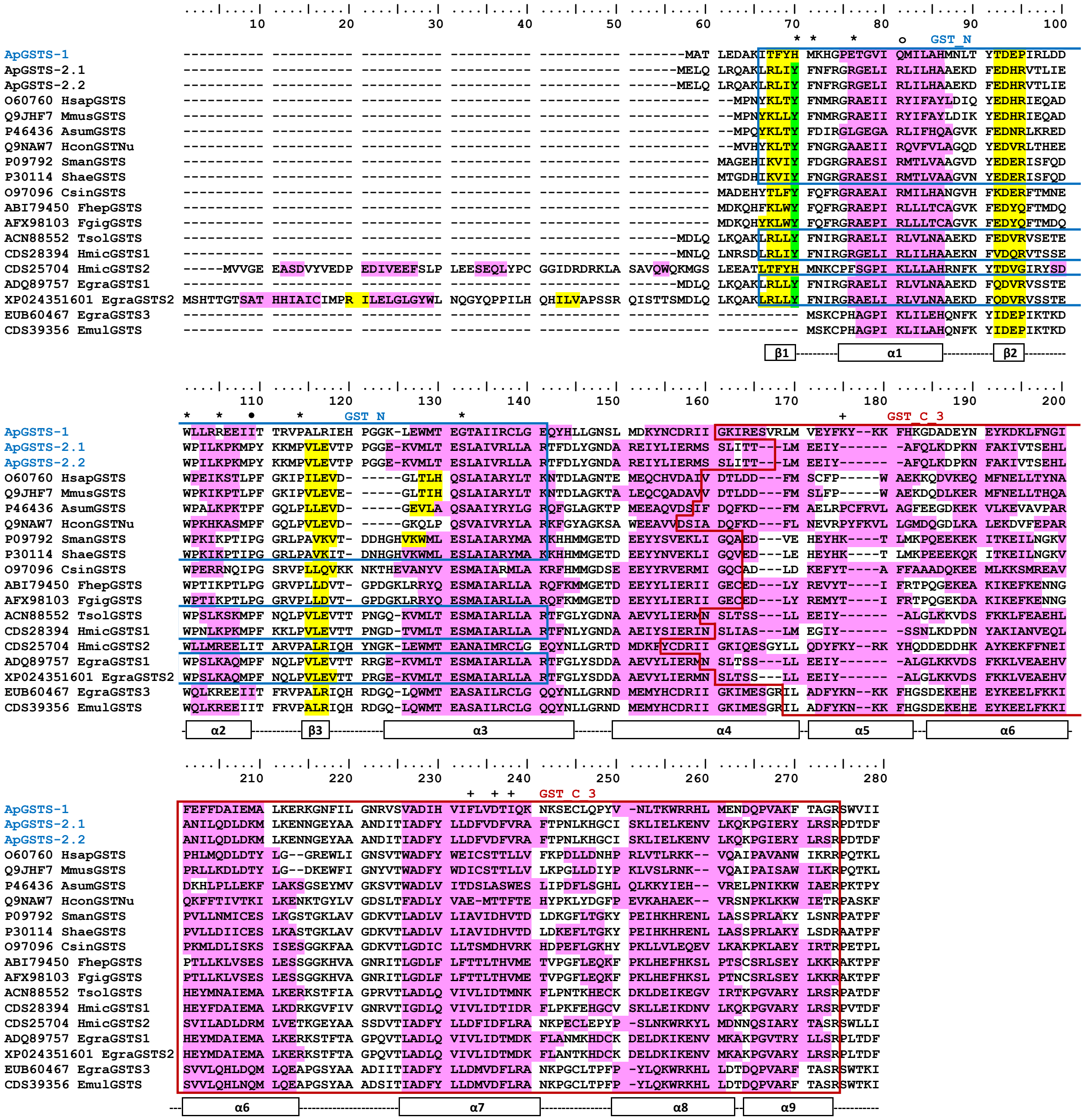

Supplementary Figure S2. Multiple sequence alignment of the two novel Sigma class GSTs identified in *A. perfoliata* (AperGST-S1 and S2). Secondary protein structure prediction using the PSIPRED Protein Analysis Workbench is presented below the alignment is comprised of 3 β-strands, shaded in yellow, and 9 α-helices, shaded in pink. The catalytic Tyrosine residues (Y) as denoted by Nguyen et al. (2010); Yamamoto et al. (2007); Zhang et al. (2020) and Xie et al. (2015) are shaded in green. The GSH-binding sites (Tyr-8, Phe-9, Arg-14, Trp-39, Lys-43, Pro-52, and Ser-64 (Nguyen et al. 2010; Zhang et al. 2020; Xie et al. 2015; Yamamoto et al. 2007) are indicated by asterisks (*) and the substrate binding sites (Nguyen et al. 2010; Zhang et al. 2020; Xie et al. 2015) are indicated by a cross (+). The predicted N- and C-terminal GST domain profiles are indicated by blue- and red-boxes
