# Supplementary material for: Evidence of Immune Modulators in the Secretome of the Equine Tapeworm Anoplocephala perfoliata
Source: Pathogens. 2021 Jul 20;10(7):912. doi: 10.3390/pathogens10070912 (PMC8308605; doi:10.3390/pathogens10070912)
Supplement: Supplementary file 1 [file pathogens-10-00912-s001.zip › Supplementary Figure S3. A. perfoliata Heat shock Protein alpha multiple sequences alignment and secondary structure prediction.pdf]

```
                          ....|....| ....|....| ....|....| ....|....| ....|....| ....|....| ....|....| ....|....| ....|....| ....|....|
                                  10         20         30         40         50         60         70         80         90        100
                                                                                                                              HATPase_c
NP_005339 HsapHSP90α      ---------- ---------- ---------- ---------- ---------- ---------M PEETQTQDQP MEEEEVETFA FQAEIAQLMS LIINTFYSNK
NP_034610 MmusHSP90α      ---------- ---------- ---------- ---------- ---------- ---------M PEETQTQDQP MEEEEVETFA FQAEIAQLMS LIINTFYSNK
Q9GKX7 EcabHSP90α         ---------- ---------- ---------- ---------- ---------- ---------M PEETHTQDQP MEEEEVETFA FQAEIAQLMS LIINTFYSNK
THD20903 FhepHSP90α       ---------- ---------- ---------- ---------- ---------- ---------M SEQMAAETVG ------ETFA FQAEIAQLMS LIINTFYSNK
TPP59511 FgigHSP90α       ---------- ---------- ---------- ---------- ---------- ---------M SEQMAAETVG ------ETFA FQAEIAQLMS LIINTFYSNK
XP_024345770 EgraHSP90α   ---------- ---------- ---------- ---------- ---------- ---------M ADQVDTDVPM AQE--VETFA FQAEIAQLMS LIINTFYSNK
CDI70178 EgraHSP90α       ---------- ---------- ---------- ---------- ---------- ---------- ---------- ---------- ---------- ----------
CDS25067 EgraHSP90        ---------- ---------- ---------- ---------- ---------- ---------M SDQEATTDDA TEQ-KVETFA FQADTRQLLN VLVNALYPTK
CDS39694 EmulHSP90        ---------- ---------- ---------- ---------- ---------- ---------M SDQEATTDNA TER-KVETFA FQADTRQLLN VLVNALYPTK
CDS28179 HmicHSP90        ---------- ---------- ---------- ---------- ---------- ---------M SD---VGCSV EVGSN----E FQGRNSQIID TTLNYLYPSE
ApHSP90-1.1               ---------- ---------- ---------- ---------- ---------- --------AM SD--QIGVNT SSGQNVEKFE FQEGALQLFD LIIKGFYPCK
ApHSP90-1.2               ---------- ---------- ---------- ---------- ---------- --AIILLRAM SN-E-SGVVT STEQNVETFE FQGEDLQLFD LIVKGFYPCK
ApHSP90-2.1               ---------- ---------- ---------- ---------- ---------- ---------M SD--QIGVNT SSGQNVEKFE FQEGALQLFD LIIKGFYPCK
ApHSP90-2.2               ---------- ---------- ---------- ---------- ---------- ---------M SD--QIGVNT SSVQNVEKFE FQEGALQLFD LIIKGFYPCK
ApHSP90-3                 ---------- ---------- ---------- ---------- ---------- ---------- LQSILFWMAC DESEGVLPWQ IQWGDRELIS LLRESVCSRN
ApHSP90-4                 ---------- ---------- ---------- ---------- ---------- ---------M TEKVET-APA NED--VETFA FQAEIAQLMS LIINTFYSNK
ApHSP90-5.1               HLINTSARGV VLRSFIDKMD HRFWWWFLVF IYLFFALINV LCADSSNDIG LDGAKPSTGT EPDLTNESLK KLIENSDEHS FSTEVPRMMK LIINSLYSHK
ApHSP90-5.2               ---------- ---------- ---------- ---------- ---------- ---------- ---------- ---------- ---------- ----------

                                                                                 ---------------------[         α1         ]------
```

```
                          ....|....| ....|....| ....|....| ....|....| ....|....| ....|....| ....|....| ....|....| ....|....| ....|....|
                                 110        120        130        140        150        160        170        180        190        200
                                                                         HATPase_c
NP_005339 HsapHSP90α      EIFLRELISN SSDALDKIRY ESLTDPSKL- DSGKELHINL IPNKQDRTLT IVDTGIGMTK ADLINNLGTI AKSGTKAFME ALQAG---AD ISMIGQFGVG
NP_034610 MmusHSP90α      EIFLRELISN SSDALDKIRY ESLTDPSKL- DSGKELHINL IPSKQDRTLT IVDTGIGMTK ADLINNLGTI AKSGTKAFME ALQAG---AD ISMIGQFGVG
Q9GKX7 EcabHSP90α         EIFLRELISN SSDALDKIRY ESLTDPSKL- DSGKELHINL IPNKQDRTLT IVDTGIGMTK ADLINNLGTI AKSGTKAFME ALQAG---AD ISMIGQFGVG
THD20903 FhepHSP90α       EIFLRELISN ASDALDKIRY KSLTDPSIL- DTCKQLEIKL IPDKANNTLT VIDTGIGMTK ADLINNLGTI ARSGTKAFME ALQTG---AD ISMIGQFGVG
TPP59511 FgigHSP90α       EIFLRELISN ASDALDKIRY KSLTDPSAL- DTGKQLEIKL IPDKANNTLT VVDTGIGMTK ADLINNLGTI ARSGTKAFME ALQTG---AD ISMIGQFGVG
XP_024345770 EgraHSP90α   EIFLRELISN GSDALDKIRY KSLTEPSVL- DTEPELCIKI IPNKAESTLT IIDTGIGMTK ADLVNNLGTI ARSGTKAFME ALNAG---AD ISMIGQFGVG
CDI70178 EgraHSP90α       ---------- ---------- ---------- ---------- ---------- -------MTK ADLVNNLGTI ARSGTKAFME ALNAG---AD ISMIGQFGVG
CDS25067 EgraHSP90        DVFLRELVSN ASDALDKLRY KSLTQPSVL- ETNAEMCIRI VPNKADATLA IMDTGVGMTK DELVNNLGRI AYSGTRAFMG ALKDGA--AD MVMAGEFGVG
CDS39694 EmulHSP90        DVFLRELVSN ASDALDKLRY KSLTQPSVL- ETNAEMCIRI VPNKADATLA IMDTGVGMTK DELVNNLGRI AYSGTRAFMG ALKDGA--AD MVMAGEFGVG
CDS28179 HmicHSP90        SVFIRELICN AFDALEKIRY LSRTDPTLIA DGDEKLGVKM ITNFPEKTLT IIDNGIGMTK KHVVEYIGVV ARSGTKEFKE QLPLDS---D ISLFGESGTG
ApHSP90-1.1               EFFIRELICN AIDALNKVRY MSLTDPSVLG ESED-LHIKI EADRTNSTLS FIDNGIGMTK MDLINNLGSV THSGTRNFKE QLSPDS---D IYLNGQYGMG
ApHSP90-1.2               EFFIRELICN AIDALNKVRY ISLTDPSILG EGEE-LHIKI ETDRASGTLS FIDNGIGMTK ADLINNLGSL THSGTRNFRE QLGPDS---G IYLNGQYGMG
ApHSP90-2.1               EFFIRELICN AIDALNKVRY MSLTDPSVLG EGED-LHIKI EADRTNGTLS FIDNGIGMTK MDLINNLGSV THSGTRNFKE QLSPDS---D IYLNGQYGMG
ApHSP90-2.2               EFFIRELICN AIDALNKVRY MSLTDPSVLG EGED-LHIKI EADRTNGTLS FIDNGIGMTK MDLINNLGSV THSGTRNFKE QLSPDS---D IYLNGQYGMG
ApHSP90-3                 ELFLVELFRN AAIALRKAGL PSLDSEK--- ---ADLFIKV APNKSEGTLT VVDNGFGMSK DDLVRNLGTL AHS--DAKFL ALNDCN---- LPQTAKSAPS
ApHSP90-4                 EIFLRELVSN GSDALDKIRY KSLTDPTVL- DTEPELCIKI IPNKADSTLT IIDTGIGMTK ADLVNNLGTI ARSGTKAFME ALNAG---AD ISMIGQFGVG
ApHSP90-5.1               EIFLRELVSN AADAVENIRF QSITNSEAL- KGLPDLAIMI KSNPAEKTIS IRDTGVGMTK GELINNLGTI AKSGTSELRE ALLNESASVS SELIGQFGVG
ApHSP90-5.2               ---------- ---------- ---------- ---------- ---------- ---------- ---------- ---------- ---------- ----------

                         -[       α2       ]--[α3]------------[ β1 ]-----[ β2 ]--------[   α4   ]-[   α5   ]-------------------------
```

```
                          ....|....| ....|....| ....|....| ....|....| ....|....| ....|....| ....|....| ....|....| ....|....| ....|....|
                                 210        220        230        240        250        260        270        280        290        300
                                                 HATPase_c                                                        HSP90
NP_005339 HsapHSP90α      FYSAYLVAEK VTVITKHNDD -EQYAWESSA GGSFTVRTDT -GEPMGRGTK VILHLKEDQ- TEYLEERRIK EIVKKHSQFI GYPITLFVEK ERDKEVSDDE
NP_034610 MmusHSP90α      FYSAYLVAEK VTVITKHNDD -EQYAWESSA GGSFTVRTDT -GEPMGRGTK VILHLKEDQ- TEYLEERRIK EIVKKHSQFI GYPITLFVEK ERDKEVSDDE
Q9GKX7 EcabHSP90α         FYSAYLVAEK VTVITKHNDD -EQYAWESSA GGSFTVRTDT -GEPMGRGTK VILHLKEDQ- TEYLEERRIK EIVKKHSQFI GYPITLFVEK ERDKEVSDDE
THD20903 FhepHSP90α       FYSAYLVADQ VKVISKNNDD -EQYIWESSA GGSFTIRPDP -GEDIGRGTK VILYFKDDQ- TEYLEEKRLK EIVKKHSQFI GYPIKLFVEK ERTKEVSDDE
TPP59511 FgigHSP90α       FYSAYLVADQ VKVISKNNDD -EQYIWESSA GGSFTIRPDP -GEAIGRGTK VILHFKEDQ- TEYLEERRLK EIVKKHSQFI GYPIKLYVEK ERTKEVSDDE
XP_024345770 EgraHSP90α   FYSAYLVADK VQVISKNNDD -EQYLWESSA GGSFTIRPCS -EEPLGRGTK VVLHIKEDQ- SEYLEERRIK EVIKKHSQFI SYPIKLFVEK ERTKEVSDDE
CDI70178 EgraHSP90α       FYSAYLVADK VQVISKNNDD -EQYLWESSA GGSFTIRPCS -EEPLGRGTK VVLHIKEDQ- SEYLEERRIK EVIKKHSQFI SYPIKLFVEK ERTKEVSDDE
CDS25067 EgraHSP90        FYSAFLVADR VEVVSKSNDD -EQYVWEWSG GESFTIRAWN -EETLKRGTK VILHMKRQQC VEYLEPHVIE AMVRKYCEFT AYPIKLCCDE EGMRSVMD--
CDS39694 EmulHSP90        FYSAFLVADR VEVVSKSNDD -EQYVWEWSG GESFTIRAWN -EETLKRGTK VILHMKRQQC VEYLEPHVIE AMVRKYCEFT AYPIKLCCDE EGMRSVMD--
CDS28179 HmicHSP90        FYTAFKAADK IQIITKHKDD -LAYVFECTD LNLYTIHPYN GEEEISRGTR VILHLNAGN- RNLLAPFMLA ELIDSHFFHA DYPIITETKW NEEDDNMI--
ApHSP90-1.1               FYSAFMVADK VQVISKHRDD -EQYMWEYAG GSSYTVSPYA -KKQLSRGTK VILHIKEKE- GNFLAGFMVS LLMDFFFTFI EYPITLEDIG VVEGRS----
ApHSP90-1.2               FYSAFMVADK VQVISKHRDD -EQYMWEYAG GSSYTVSPYA -KKQLSRGTK VILHIKEKE- GNFLAGFMVS LLMDFFFTFI EYPITLEDIG VVEGRS----
ApHSP90-2.1               FYSALMVADR IQVISKHKDD -EQFMWEYAG GNSYTVSPYN -QEQLGRGTK VILHIKEGE- GSVLVSFMVA LLMDFFFEFI DYPITLEDIG VEQSSSSD--
ApHSP90-2.2               FYSALMVADR IQVISKHKDD -EQFMWEYAG GNSYTVSPYN -QEQLGRGTK VILHIKEGE- GNVLVAFMVA LLMDFFFEFI DYPITLEDIG VEQSSSSD--
ApHSP90-3                 FQSIFRVADK VQVTSKTDAS GSQFFFEFHS GKPFTVRSCD -EENLDRGTK VVLHLKKKY- ADFLLSSVLE DFIHKYVEFI GFPIKLISYK EGGAYEER--
ApHSP90-4                 FYSAYLVADK VQVISKNNDD -EQYLWESSA GGSFTIRPYN -EEPLGRGTK VILHIKEDQ- SEYLEERRLK EVVKKHSQFI SYPIKLYIEK ERTKEVPDED
ApHSP90-5.1               FYSAFLVADR VTVISKHNDD -DQHVWESDS -TSFKIAKDP RGNTLGRGTE VIIHVKEEA- KEYLQNDVLE KILQKYSQFV TCPIKLWKS- -RTESVEEEV
ApHSP90-5.2               ---MHLVADR VTVISKHNDD -DQHVWESDS -TSFKIAKDP RGNTLGRGTE VIIHVKEEA- KEYLQNDVLE KILQKYSQFV TCPIKLWKS- -RTESVEEEV

                         -[α6]------[  β3  ]-----------[ β4 ]-----[ β5  ]------------[  β6  ]-------[α7]-[   α8   ]----------[β7]---------------
```

Supplement Figure S3. Multiple sequence alignment of the 5 (including isoforms of each) novel *A. perfoliata* Heat Shock Protein 90 alpha (HSP90 alpha/α) sequences with recognised HSP90 alpha isoforms BLAST hits sequences. The secondary protein structure prediction the HSP90 alpha characteristic structure using the PSIPRED Protein Analysis Workbench (presented below the alignment). Each β-strand or α-helix is numbered and shaded in yellow (β-strand) and pink (α-helix). The domain analysis of C-terminal and N-terminal domain of HSP90 alpha isoforms using SMART tools is boxed in blue and red, respectively. Unique cytosolic HSP90, MEEVD peptide sequences are in green type and the unique cytosolic HSP90 alpha, LIP and EDD peptide sequences are shaded in green (Chen et al., 2006; Skantar & Carta, 2004).

```
                          ....|....| ....|....| ....|....| ....|....| ....|....| ....|....| ....|....| ....|....| ....|....| ....|....|
                                310        320        330        340        350        360        370        380        390        400
                                                                               HSP90
NP_005339 HsapHSP90α      AEEKEDKEEE KEKEEKESED KPEIEDVGSD EEEE-KKDGD --------KK KKKKIKEKYI DQEELNKTKP IWTRNPDDIT NEEYGEFYKS LTN-DWEDHL
NP_034610 MmusHSP90α      AEEKEEKEEE KEKEEKESDD KPEIEDVGSD EEEEEKKDGD --------KK KKKKIKEKYI DQEELNKTKP IWTRNPDDIT NEEYGEFYKS LTN-DWEEHL
Q9GKX7 EcabHSP90α         AEEKEDKEEE KEKEEKESDD KPEIEDVGSD EEEEEKKDGD --------KK KKKKIKEKYI DQEELNKTKP IWTRNPDDIT NEEYGEFYKS LTN-DWEDHL
THD20903 FhepHSP90α       EE----VEKK EETKEDEKDD KPKVEDLDED E-EDGDKSES ---------K KKKKVTEKYV DEEELNKTKP LWTRNPDDIT QEEYGSFYKQ LSN-DWEDHL
TPP59511 FgigHSP90α       EEE---GEKK GDTKEDEKDD KPKVEDLDED EDEDGDKSGS ---------K KKKKVTEKYV DEEELNKTKP LWTRNPDDIT QEEYGSFYKQ LSN-DWEDHL
XP_024345770 EgraHSP90α   EEEVK-EEEK ADAEK-PDVE KPEVEDLDEE ESEEEGGEGS -GEKSEKKKK KKKQIKEKYI DEEELNKTKP LWTRNPEDIS QEEYGEFYKS LSN-DWEDHL
CDI70178 EgraHSP90α       EEEVK-EEEK ADAEK-PDVE KPEVEDLDEE ESEEEGGEGS -GEKSEKKKK KKKQIKEKYI DEEELNKTKP LWTRNPEDIS QEEYGEFYKS LSN-DWEDHL
CDS25067 EgraHSP90        ---------- ---------- ---CEEWRRD EE-------- ---------- --------VV DGVVLNRMEP LWMRSAEDIS SEEYGKFYKW LSC-DSEDHM
CDS39694 EmulHSP90        ---------- ---------- ---REEWRRD GE-------- ---------- --------VV DGVVLNRMEP LWMRSAEDIS SEEYGKFYKW LSC-DSEDHM
CDS28179 HmicHSP90        ---------- ---------- ---------- ---------- ---------- ---------- -QPLKHMTNH VWSNDFDVLT EGEINKLYEE ISFNKNS-HI
ApHSP90-1.1               ---------- ---------- ---------- ---------- ---------- ---------- ---EYERRRP LWMYEPEEIE EEDYRKLLGW MTLDEASSYL
ApHSP90-1.2               ---------- ---------- ---------- ---------- ---------- ---------- ---EYERRRP LWMYEPEEIE EEDYRKLLGW MTLDEASSYL
ApHSP90-2.1               ---------- ---------- ---------- ---------- ---------- ---------- -YLKYKRRKP LWMYEPEEVE EEDYKKLLGW MTLNETSSYL
ApHSP90-2.2               ---------- ---------- ---------- ---------- ---------- ---------- -YLKYKRRKP LWMYEPEEVE EEDYKKLLGW MTLNETSSYL
ApHSP90-3                 ---------- ---------- ---------- ---------- ---------- ---------- ---LINDVPS VWMHNPEEVR HEDYVELYKY LSD-DTEEPL
ApHSP90-4                 AVEEE-EEKK EEGEKKEEGE KPEVEDLDED ESDDEGGEDE KGEKKTKKKK KTKQIKEKYI DVEELNKTKP LWTRNAEDIS KEEYAEFYKS LSN-DWEDHL
ApHSP90-5.1               EEEAK----- ---------- SDTSDDVSVE EEKP------ ---------K EKKTVEKTVW DWVQLNGQKP LWTKKPSEVA DEEYEELFKL LSR-STEAPL
ApHSP90-5.2               EEEAK----- ---------- SDTSDDVSVE EEKP------ ---------K EKKTVEKTVW DWVQLNGQKP LWTKKPSEVA DEEYEELFKL LSR-STEAPL
                          -[   α9   ]---------------------------------------------------[ β8 ]--------------------[α10]---[  α11  ]----------

                          ....|....| ....|....| ....|....| ....|....| ....|....| ....|....| ....|....| ....|....| ....|....| ....|....|
                                410        420        430        440        450        460        470        480        490        500
                                                                               HSP90
NP_005339 HsapHSP90α      AVKHFSVEGQ -LEFRALLFV PRRAPFDLFE N-RKKKNNIK LYVRRVFIMD NCEELIPEYL N-FIRGVVDS EDLPLNISRE MLQQSKILKV IRKNLVKKCL
NP_034610 MmusHSP90α      AVKHFSVEGQ -LEFRALLFV PRRAPFDLFE N-RKKKNNIK LYVRRVFIMD NCEELIPEYL N-FIRGVVDS EDLPLNISRE MLQQSKILKV IRKNLVKKCL
Q9GKX7 EcabHSP90α         AVKHFSVEGQ -LEFRALLFV PRRAPFDLFE N-RKKKNNIK LYVRRVFIMD NCEELIPEYL N-FIRGVVDS EDLPLNISRE MLQQSKILKV IRKNLVKKCL
THD20903 FhepHSP90α       AVKHFSVEGQ -LEFRALLFV PKRAPFDLFE G-RKKRNNIR LYVRRVFIMD NCEELIPEYL N-FVKGVVDS EDLPLNISRE MLQQNKILKV IRKNLVKKCL
TPP59511 FgigHSP90α       AVKHFSVEGQ -LEFRALLFV PKRAPFDLFE G-RKKRNNIR LYVRRVFIMD NCEELIPEYL N-FVKGVVDS EDLPLNISRE MLQQNKILKV IRKNLVKKCL
XP_024345770 EgraHSP90α   AVKHFSIEGQ -LEFRALLFV PKRAPFDLFE N-RKKRNNIK LYVRRVFIMD NCEDLIPEYL N-FIKGVVDS EDLPLNISRE MLQQNKILKV IRKNLVKKCM
CDI70178 EgraHSP90α       AVKHFSIEGQ -LEFRALLFV PKRAPFDLFE N-RKKRNNIK LYVRRVFIMD NCEDLIPEYL N-FIKGVVDS EDLPLNISRE MLQQNKILKV IRKNLVKKCM
CDS25067 EgraHSP90        AVKHFSVEGE -LSFRALLFV PRRPPAAYFA R-NGRRHNIR LYVKRVLVTE ECESLIPDYL N-FLIGVVDS EDLPLNLSRE AIRESSVVRA IGKHLVRKSI
CDS39694 EmulHSP90        AVKHFSVEGE -LSFRALLFV PRRPPAAYFA R-NGRPHNIR LYVKRVLVTE ECESLIPDYL N-FLIGVVDS EDLPLNLSRD AIRESSVVRA IGKHLVRKSI
CDS28179 HmicHSP90        FVKHIKYHEA SISFEALIYV PKKDPHFCTW N--TGEYNVH FIWKGLKLPK RKECLLPTYM D-FVSVIVNV DTKLLNANRT DLQG-HLRQE IGQVLVENVL
ApHSP90-1.1               LVKHIKYKDS -ISFRALIFV PKLGPIDFIL D--SKPRHVR LFSQHIQITE CSKGLLPECM N-FVSVIVDS DDVLLNANRS DFHDPKVKQL ISEVLAEKVF
ApHSP90-1.2               LVKHIKYKDS -ISFRALIFV PKLGPVDFIL D--SKPRHVR LFSQHIQITE CSKGLLPECM N-FVSVIVDS DDVLLNANRS DFHDPKEK-- ----------
ApHSP90-2.1               LVKHIKYKGL -ISFRALIFV PRVGPVDFSL V--CKPPKVR LFSQRIQITQ CSEGLLPEFM N-FVSVIVDS DNVLLNANRS DFHDLKEKQL ISEVLTDNVF
ApHSP90-2.2               LVKHIKYKGL -ISFRALIFV PRVGPVDFSL V--CKPPKVR LFSQRIQITQ CSEDLLPEFM N-FVSVIVDS DNVLLNANRS DFHDLKEKQL ISEVLTDNVF
ApHSP90-3                 YTKHFSCPGE -TSMRGVLFI PKQYHKDTAQ H------GVE VYLNQVLLTD SLETLFEGFP HDHLYGVIDL FNLHLTSDCS DSVLRKGKKE IYAHLREVFL
ApHSP90-4                 AVKHFSIEGQ -LEFRALLFV PKRAPFDLFE N-RKKRNNIK LYVRRVFIMD NCEDLIPEYL N-FIRGVVDS EDLPLNISRE MLQQNKILKV IRKNLVKKCI
ApHSP90-5.1               AHIHFSAEGE -VNFDAVLFV SKTPRPDLFQ GDKAITDNVK LYVRRVFITE NVDELLPRYL G-FVYGIVDS DNLPLNVSRE MLQQHKLLKL IKKKLVRKVL
ApHSP90-5.2               AHIHFSAEGE -VNFDAVLFV SKTPRPDLFQ GDKAITDNVK LYVRRVFITE NVDELLPRYL G-FVYGIVDS DNLPLNVSRE MLQQHKLLKL IKKKLVRKVL
                          [ β9 ]-[   β10   ]----------------------------[  β11  ]---[α12]-[ α13 ]-[β12]----------[ α14 ]--[    α14    ]-

                          ....|....| ....|....| ....|....| ....|....| ....|....| ....|....| ....|....| ....|....| ....|....| ....|....|
                                510        520        530        540        550        560        570        580        590        600
                                                                               HSP90
NP_005339 HsapHSP90α      ELFTELAEDK ENYKKFYEQF SKNIKLGIHE DSQNRKKLSE LLRYYTSASG DEMVSLKDYC TRMKENQKHI YYITGETKDQ VANSAFVERL RKHGLEVIYM
NP_034610 MmusHSP90α      ELFTELAEDK ENYKKFYEQF SKNIKLGIHE DSQNRKKLSE LLRYYTSASG DEMVSLKDYC TRMKENQKHI YFITGETKDQ VANSAFVERL RKHGLEVIYM
Q9GKX7 EcabHSP90α         ELFTELAEDK ENYKKFYEQF SKNIKLGIHE DSQNRKKLSE LLRYYTSASG DEMVSLKDYC TRMKENQKHI YYITGETKDQ VANSAFVERL RKHGLEVIYM
THD20903 FhepHSP90α       ELFEEIMEDK DNYATFYGQF AKNLKLGIHE DSVHRKKIAE FLRYYSTSSP DELTSLKDYV SRMKENQKDI YYITGESKAA VSDSAFMEVL RKRGYEVLYM
TPP59511 FgigHSP90α       ELFEEIMEDK DNYATFYGQF AKNLKLGIHE DSVHRKKLAE FLRYYSTSSP DELISLKDYV SRMKENQKDI YYITGESKAA VSDSAFMEVL RKRGYEVLYM
XP_024345770 EgraHSP90α   ELFEELAEDT ETYKKFYEQF SKNIKLGIHE DSTNRKKLAE LLRYYSSQSG DEMTSLKDYV SRMKEHQKDI YYITGESKES LANSAFIEVL KKRGLEVLYM
CDI70178 EgraHSP90α       ELFEELAEDT ETYKKFYEQF SKNIKLGIHE DSTNRKKLAE LLRYYSSQSG DEMTSLKDYV SRMKEHQKDI YYITGESKES LANSAFIEVL KKRGLEVLYM
CDS25067 EgraHSP90        ELMQEMAEDA QAYSAFYGNF QRSIKLGVYE DRGHRQKLAN LLRYYSSKSG DEMIGLREYV CRMKEGQSDI YCMIGESMAS VSDSVFMEGL KKRDVEVLLM
CDS39694 EmulHSP90        ELMQEMAEDA QAYSAFYGNF QRSIKLGVYE DRGHRQKLAN LLRYYSSKSG DEMIGLREYV CRMKEGQSDI YCMIGESMAS VSDSVFMEGL KKRDVEVLLM
CDS28179 HmicHSP90        NAFSEIAEDP EMFDLFHNQY YKNIEYGVLS DSDNCQKLLA LMGFYTLKNK EKAIRMCDYV EKMKPDQKEI YYLIEGDEKP TSNSPFLEHL RKRDYDAILI
ApHSP90-1.1               EILDEIVEKP EIFNEFHKYY SEDIKISVKT DEEYGHRLLP FMSFYTLNNG NKAILLKDYV AQMKPDQQEI YYLAGEDLKI LSNSPLLENL KKRGFDVILM
ApHSP90-1.2               ---------- ---------- ---------- ---------- ---------- ---------- ---------- ---------- ---------- ----------
ApHSP90-2.1               EILNEIVKKP ETFNEFHKYY SESIKLGVKT DKKYFQRLLP FMSFYTLKNG HKATLLKDYV TKMKQDQQVI YYLVGEDLKI LSNSPLLENF KKRDLDVILM
ApHSP90-2.2               EILNEIVKKP ETFNEFHKYY SESIKLGVKT DKKYFQRLLP FMSFYTLKNG HKATLLKDYV TKMKQDQQVI YYLVGEDLKI LSNSPLLENF KKRDLDVILM
ApHSP90-3                 EWFKAISESE DVYCAVCENL KPLLLFLYRT DAR----FAG FLRYYSSKSG NLRISLDEYI SRMKEDQRVI YIALGDNVEM LRDLPAAEKL HQSGIEVLYT
ApHSP90-4                 ELFEELAEDP ETYKKFYEQF SKNIKLGIHE DSTNRKKLAD LLRYYSSLSG DEMTSLKDYV SRMKENQKDI YYITGESKES MVDSSFVEVL KKRGLEVLYM
ApHSP90-5.1               EMLQKMP--K EDYEKFWKKY AVAMKLGILQ DMPNKSKLTD LLRFYTSKSA DDLTSFNEYM SRMKEGQEEI YYITGQSIME VKNSLFVERI QRLGYEVIYF
ApHSP90-5.2               EMLQKMP--K EDYEKFWKKY AVAMKLGILQ DMPNKSKLTD LLRFYTSKSA DNLTSFNEYM SRMKEGQEEI YYITGQSINE VKNSLFVERI QRLGYEVIYF

                          [ α14 ]-[    α15    ]---------[  α16  ]-----------------[ α17 ]--------[ β12 ]---[α18]--[ α19 ]----[β13]-
```

Supplement Figure S3-Continued 2. Multiple sequence alignment of the 5 (including isoforms of each) novel *A. perfoliata* Heat Shock Protein 90 alpha (HSP90 alpha/α) sequences with recognised HSP90 alpha isoforms BLAST hits sequences. The secondary protein structure prediction the HSP90 alpha characteristic structure using the PSIPRED Protein Analysis Workbench (presented below the alignment). Each β-strand or α-helix is numbered and shaded in yellow (β-strand) and pink (α-helix). The domain analysis of C-terminal and N-terminal domain of HSP90 alpha isoforms using SMART tools is boxed in blue and red, respectively. Unique cytosolic HSP90, MEEVD peptide sequences are in green type and the unique cytosolic HSP90 alpha, LIP and EDD peptide sequences are shaded in green (Chen et al., 2006; Skantar & Carta, 2004).

```
                              ....|....| ....|....| ....|....| ....|....| ....|....| ....|....| ....|....| ....|....| ....|....| ....|....|
                                     610        620        630        640        650        660        670        680        690        700
                                                                                  HSP90
NP_005339 HsapHSP90α          IEPIDEYCVQ QLKEFEGKTL VSVTKEGLEL PED------- -----EEEKK KQEE------ KKTKFENLCK IMKDI-LEKK VEKVVVSNRL VTSPCCIVTS
NP_034610 MmusHSP90α          IEPIDEYCVQ QLKEFEGKTL VSVTKEGLEL PED------- -----EEEKK KQEE------ KKTKFENLCK IMKDI-LEKK VEKVVVSNRL VTSPCCIVTS
Q9GKX7 EcabHSP90α             IEPIDEYCVQ QLKEFEGKTL VSVTKEGLEL PED------- -----EEEKK KQEE------ LKTKFENLCK IMKDI-LEKK VEKVVVSNRL VTSPCCIVTS
THD20903 FhepHSP90α           LDPIDEYAVT QLREYDGKKL VCVTKEGLQL PED------- -----EEEKK EFEE------ LKAAYEPLCK QIQEI-LGKR VEKVAVSNRM TSSPCCIVTS
TPP59511 FgigHSP90α           LDPIDEYAVT QLREYDGKKL VCVTKEGLQL PED------- -----EEEKK EFEE------ LKAAYEPLCK QIQEI-LGKR VEKVAVSNRM TSSPCCIVTS
XP_024345770 EgraHSP90α       VDPIDEYAVP QLREYDGKKL VCVTKEGLEL PED------- -----EEEKK RFEE------ VKASFEPTCK KIKDI-LGNK VEKVTVSNRL TTSPCCIVTS
CDI70178 EgraHSP90α           VDPIDEYAVP QLREYDGKKL VCVTKEGLEL PED------- -----EEEKK RFEE------ VKASFEPTCK KIKDI-LGNK VEKVTVSNRL TTSPCCIVTS
CDS25067 EgraHSP90            VDPMDEYVVN AMTEYLGKSL VCVSRLDLQL PEEKVEAVVA E-----EEGK TTTQGEWMEC VVADFESTCG KMKEI-LGER VESVRVSSRL TTSPCCVVTS
CDS39694 EmulHSP90            VDPMDEYVVN AMTEYLGKSL VCVSRLDLQL PEERVEAVVA EAAAAEEERK TTTQGEWMEC VVADFEATCG KMKEI-LGER VESVRVSSRL TTSPCCVVTS
CDS28179 HmicHSP90            QGETRQRIIG MVISYEDMEL VDVSKMTREP ENNLN----- ------EDYS KILD------ ---EYRFMLE KIKEL-LGDK IVDLKISQVP LDVPCCIST-
ApHSP90-1.1                  DTVIDQYIAD LIVSYADKKL VDVSATNFEM STDLEQ---- -----DENDL RISS------ ---EFESTCK SVRAI-LADQ VEGVIVSNKL CDAPCCISAS
ApHSP90-1.2                  ---------- ---------- ---------- ---------- ---------- ---------- ---------- ---------- ---------- ----------
ApHSP90-2.1                  NTKIDQSIVD MIVSYDNKEL VDVSATNLEM LNDLEQ---- -----KENDK RISS------ ---EFESTCK SIKKI-LANE VEDVVVSNKI SYTPCCISTS
ApHSP90-2.2                  NTKIDQSIVD MIVSYDNKEL VDVSATSLEM LNDLEQ---- -----KENDK RISS------ ---EFESTCK SIKKI-LANE VEDVVVSNKI SYTPCCISTS
ApHSP90-3                    SNPADYSFIC DKNCYKLYKI RSVGSYANKI ELS------- -----KEEER EYRES----- -CVEFASTCS TMENY-YEGG HASVRVSRRL VNSPCCAFVG
ApHSP90-4                    CDPIDEYAVT QLREYDGKKL VCVTKEGLEL PED------- -----DEEKK RFEE------ VKAAFEPTCK KIKDI-LGNK VEKVSVSNKL VSSPCCIVTS
ApHSP90-5.1                  TEPMDEYVMQ ALSEVKGKKT QNLAKGGLDL KLE------- -----KESKE KFEE------ QERAFKPLTD WLRDKGLSGK IKDAKISQRL DSSPCALVAD
ApHSP90-5.2                  TEPMDEYVMQ ALSEVKGKKT QNLAKGGLDL KLE------- -----KESKE KFEE------ QERAFKPLTD WLRDKGLSGK IKDAKISQRL DSSPCALVAD

                             ------[ α20 ]----------[ β14 ]------------------------------[                α21                ]--[ β15 ]----------[ β16 ]-

                              ....|....| ....|....| ....|....| ....|....| ....|....| ....|....| ....|....| ....|....| ....|....| ....|....|
                                     710        720        730        740        750        760        770        780        790        800
                                                                                  HSP90
NP_005339 HsapHSP90α          TYGWTANMER IMKAQALR-- -DNSTMGYMA AKKHLEINPD HSIIETLRQK AEA-DKNDKS VKDLVILLYE TALLSSGFSL EDPQTHANRI YRMIKLGLGI
NP_034610 MmusHSP90α          TYGWTANMER IMKAQALR-- -DNSTMGYMA AKKHLEINPD HSIIETLRQK AEA-DKNDKS VKDLVILLYE TALLSSGFSL EDPQTHANRI YRMIKLGLGI
Q9GKX7 EcabHSP90α             TYGWTANMER IMKAQALR-- -DNSTMGYMA AKKHLEINPD HSIIETLRQK AEA-DKNDKS VKDLVILLYE TALLSSGFSL EDPQTHANRI YRMIKLGLGI
THD20903 FhepHSP90α           EFGWSANMER IMKAQALR-- -DSSTMGYMA AKKHLELNPR HKIVQSLKSL FEK-GESNKL AKDLVFLLYS TSLLSSGFSL EDPKVHSGRI HQLISMCLDI
TPP59511 FgigHSP90α           EFGWSANMER IMKAQALR-- -DSSTMGYMA AKKHLELNPR HKIVQSLKSL FEK-GESNKL AKDLVFLLYS TALLSSGFSL EDPKVHSGRI HQLISMCLDI
XP_024345770 EgraHSP90α       TFGWSANMER IMKAQALR-- -DSSTMGYMS AKKHLEINPD HKVMIRLKEM LSAEGEPNKI CKDLINMLFS TALLASGFTL EDPKAHANKI HELISMCLEI
CDI70178 EgraHSP90α           TFGWSANMER IMKAQALR-- -DSSTMGYMS AKKHLEINPD HKVMIRLKEM LSAEGEPNKI CKDLINMLFS TALLASGFTL EDPKAHANKI HELISMCLEI
CDS25067 EgraHSP90            TFGWSANMQR IGKAQALR-- -NPHSLRNNS AKKHLEINAN DSIIIGLKKM LSTEGMPNKI SRDMLEILYN TALLDSGFVL KEPKTHTNTI HALIRMFLEI
CDS39694 EmulHSP90            TFGWSANMQR IGKAQALR-- -NPHSLRNNS AKKHLEINAN DSIIIGLKKM LSTEGMPNKI SRDMLEILYN TALLDSGFVL EEPKTHTNTI HALIRMFLEI
CDS28179 HmicHSP90            VEEVEVN--- ---------- ---------- --KILIINHK DPIINYLKES IRKMENSDIL WKNIATLLYS TAVIRSGNIL ENCRDLPKLM FRFVNKYLED
ApHSP90-1.1                  TEGISLNMAR ILKSQ-RG-- -GDSSTEKPA SKKYFEINAK HPMINWLKKT LDQEESPSTA SKNLVNMLYS AALLRSGNTL ERPRSFTKLM YSFINMYLKE
ApHSP90-1.2                  ---------- ---------- ---------- ---------- ---------- ---------- ---------- ---------- ---------- ----------
ApHSP90-2.1                  TEDASLNRVS ILKSPGRG-- -DSSSTKKPA SKKYFEINPK DPIINRLKKM LDQGESPNTS SKNIVNMLYS AALLRSGKTL ERPHSFTKLM YNFVNMCLKE
ApHSP90-2.2                  TEDASLNRVS ILKSPGRG-- -DSSSTKKPA SKKYFEINPK DPIINRLKKM LDQGESPNTS SKNIVNMLYS AALLRSGNTL ERPRSFTKLM YSFINMYLKE
ApHSP90-3                    --------GK LPFGEPLK-- ---PSSPREE EEVNFEINPN HEVLIRLKEL CCNNDGNLNA YAPIINILLE TALIDSGFMP PDPIRYADMV YELIGASLNV
ApHSP90-4                    TFGWSANMER IMKAQALR-- -DSSTMGYMS AKKHLEINPD HKIMIRLKEM LSADGEPNKI CKDLINMLFS TALLTSGFTL EDPKAHANKI HELISMCLEI
ApHSP90-5.1                  EYMASGNFQK VMLSQAYGSI DPATNAYYLN QRQTLEVNPR HPLIKKLNEL VQN-DPNDEV AKENTMLLYD TAVLRSGFIM QDLVGFAERI ERELRKNLDV
ApHSP90-5.2                  EYMASGNFQK VMLSQAYGSI DPATNAYYLN QRQTLEVNPR HPLIKKLNEL VQN-DPNDEV AKENTMLLYD TAVLRSGFIM QDLVGFAERI ERELRKNLDV
                             -------[    α22    ]-------------------------[ β17 ]------[    α23    ]--------[      α24      ]------------[     α25     ]----

                              ....|....| ....|....| ....|....| ....|....| ....|
                                     810        820        830        840
                                        HSP90
NP_005339 HsapHSP90α          DEDDPTA--D DTSAAVTEE- --MPPLEGDD DTSRMEEVD- -----
NP_034610 MmusHSP90α          DEDDPTV--D DTSAAVTEE- --MPPLEGDD DTSRMEEVD- -----
Q9GKX7 EcabHSP90α             DEDDPTA--D DSSAAVTEE- --MPPLEGDD DTSRMEEVD- -----
THD20903 FhepHSP90α           PTDDEMK--T DTPEVPSST- --ATPAEAGD D-AGMEEVD- -----
TPP59511 FgigHSP90α           PTDDEMK--T DTPEVPSST- --ATPAEAGD D-AGMEEVD- -----
XP_024345770 EgraHSP90α       PEDETMK--E EEKNTASSD- -VAAPVEAGD DGAAMEEVD- -----
CDI70178 EgraHSP90α           PEDETMK--E EEKNTASSD- -VAAPVEAGD DGAAMEEVD- -----
CDS25067 EgraHSP90            PECESVK--D AEQVTADSD- -EAALVQAGD DGGAMGEVG- -----
CDS39694 EmulHSP90            PECESVK--D AEQVTADSD- -EAALVQAGD DGGAMGEVG- -----
CDS28179 HmicHSP90            VKLTSVSDAE ------SKDE LNYEIDSSPM NRLA------ -----
ApHSP90-1.1                  HKEIMKATKE GIDEERSPGE FNFEIDSCPM NVLL------ -----
ApHSP90-1.2                  ---------- ---------- ---------- ---------- -----
ApHSP90-2.1                  QKEIKVATKE LLNENRSPKE FSLGINHCPK IVRKEKKSSW VRIYF
ApHSP90-2.2                  HKEIMKATKE GIDEERSPGE FNFEIDSCPM NVLL------ -----
ApHSP90-3                    SKTTEMPKET EERALYESET --SSPILAAD KGGDVDCTP- -----
ApHSP90-4                    PEDETMKDVS EEKANATTEG GATAPIEAGD DGAAMEEVD- -----
ApHSP90-5.1                  DVAEQVEPDD SIPEPVEDEK -KEESSEQAE PMDVKEEL-- -----
ApHSP90-5.2                  DVAEQVEPDD SIPEPVEDEK -KEESSEQAE PMDVKEEL-- -----
```

Supplement Figure S3-Continued 3. Multiple sequence alignment of the 5 (including isoforms of each) novel *A. perfoliata* Heat Shock Protein 90 alpha (HSP90 alpha/α) sequences with recognised HSP90 alpha isoforms BLAST hits sequences. The secondary protein structure prediction the HSP90 alpha characteristic structure using the PSIPRED Protein Analysis Workbench (presented below the alignment). Each β-strand or α-helix is numbered and shaded in yellow (β-strand) and pink (α-helix). The domain analysis of C-terminal and N-terminal domain of HSP90 alpha isoforms using SMART tools is boxed in blue and red, respectively. Unique cytosolic HSP90, MEEVD peptide sequences are in green type and the unique cytosolic HSP90 alpha, LIP and EDD peptide sequences are shaded in green (Chen et al., 2006; Skantar & Carta, 2004).
