# Supplementary material for: Evidence of Immune Modulators in the Secretome of the Equine Tapeworm Anoplocephala perfoliata
Source: Pathogens. 2021 Jul 20;10(7):912. doi: 10.3390/pathogens10070912 (PMC8308605; doi:10.3390/pathogens10070912)
Supplement: Supplementary file 1 [file pathogens-10-00912-s001.zip › Supplementary Figure S5. Protein profiles of A. perfoliata.pdf]

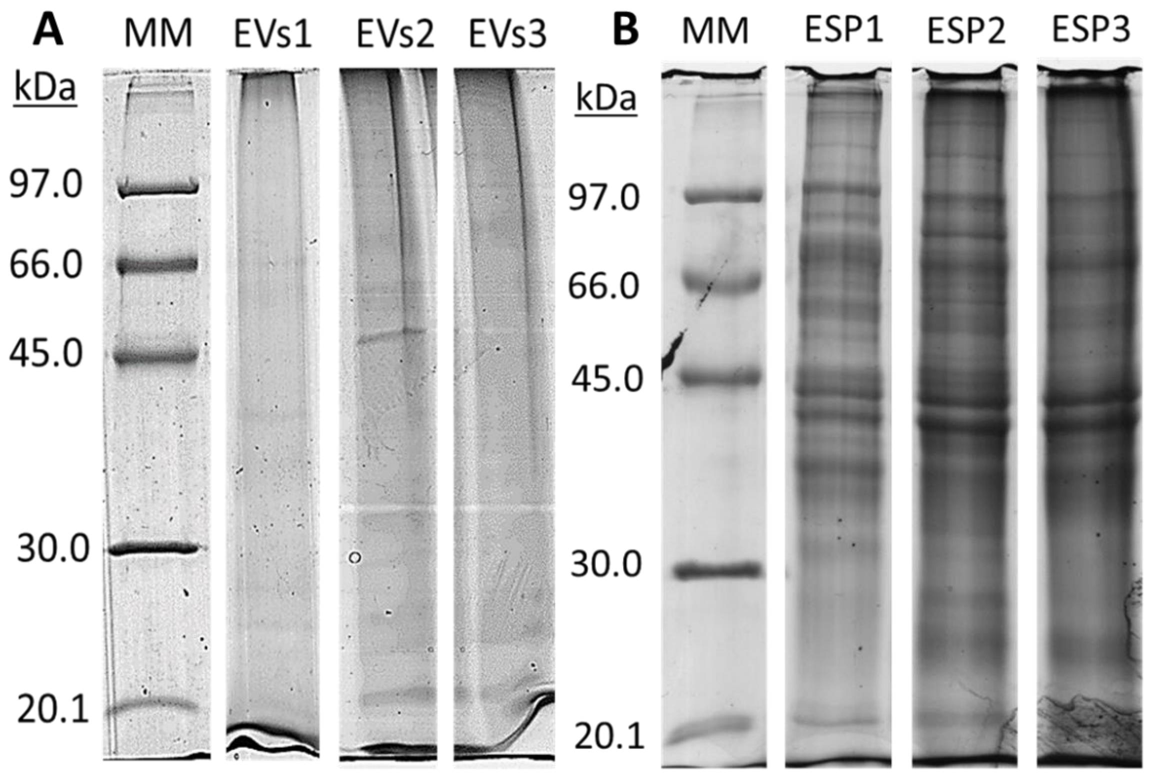

Supplementary Figure S5. Protein profiles of the *A. perfoliata* secretome fractions. (A) whole extracellular vesicles (EVs) and (B) EV depleted excretory/secretory protein (ESP) from 3 biological replicates displayed on a 12.5% one-dimension sodium dodecyl sulphate polyacrylamide gel electrophoresis (1D SDS-PAGE) gel stained with colloidal Coomassie blue. A total of 10 µg of each protein sample was loaded per lane. MM: Molecular weight markers.
