# Supplementary material for: Evidence of Immune Modulators in the Secretome of the Equine Tapeworm Anoplocephala perfoliata
Source: Pathogens. 2021 Jul 20;10(7):912. doi: 10.3390/pathogens10070912 (PMC8308605; doi:10.3390/pathogens10070912)
Supplement: Supplementary file 1 [file pathogens-10-00912-s001.zip › Supplementary Figure S6. Gene enrichment analysis of A perfoliata proteomics datasets.pdf]

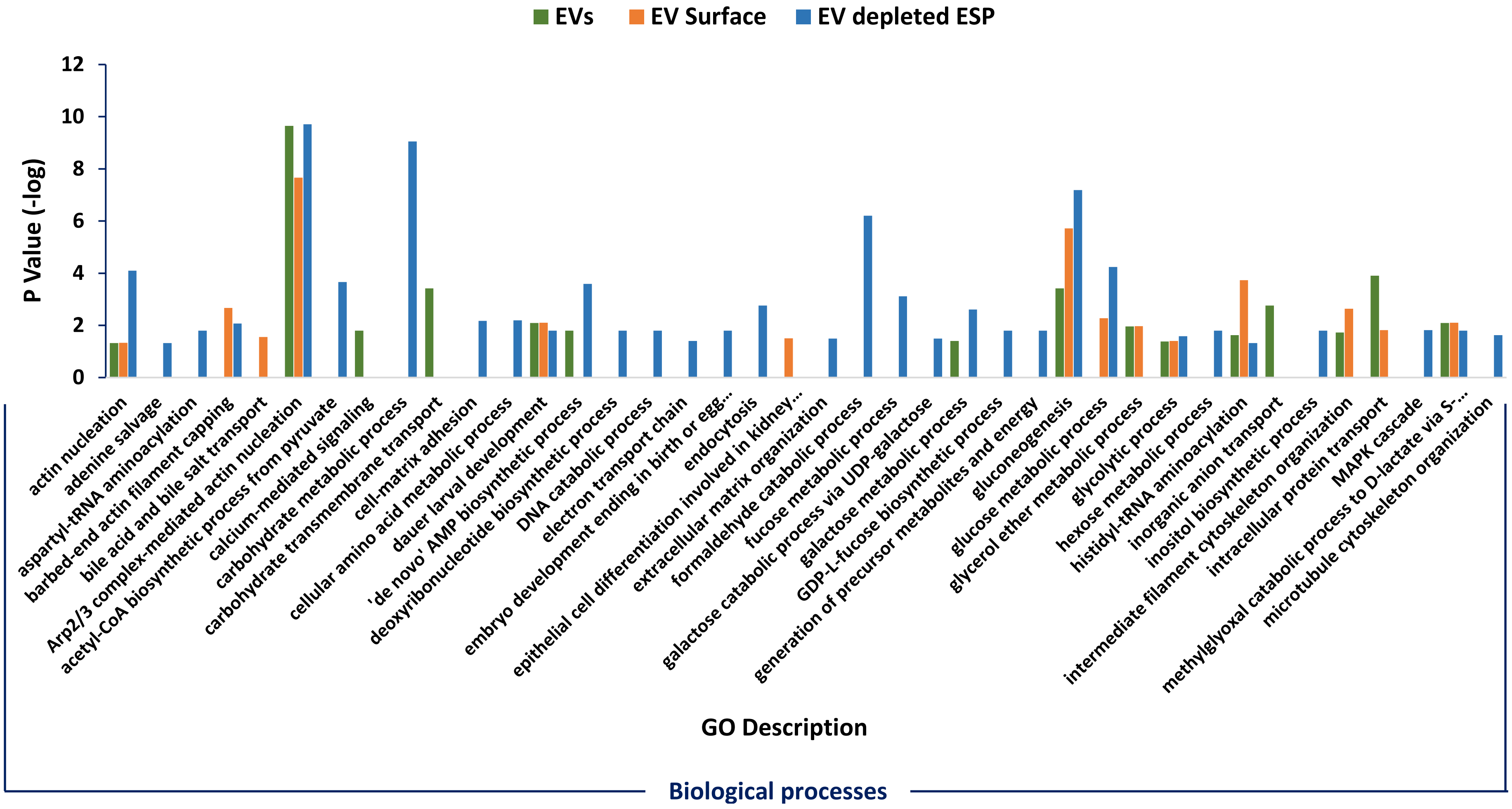

Supplementary Figure S6. Gene ontology enrichment analysis using GOATOOLS in python comparing all 3 proteomic datasets from the *A. perfoliata* secretome compared to the whole *A. perfoliata* transcriptome for the Biological process functional category. GO terms were not propagated up the hierarchy (nop) ($p<0.05$ identified significance) and where enriched in all or any of the 3 proteomic datasets were presented using -log transformation of p_uncorrected value and GO description.

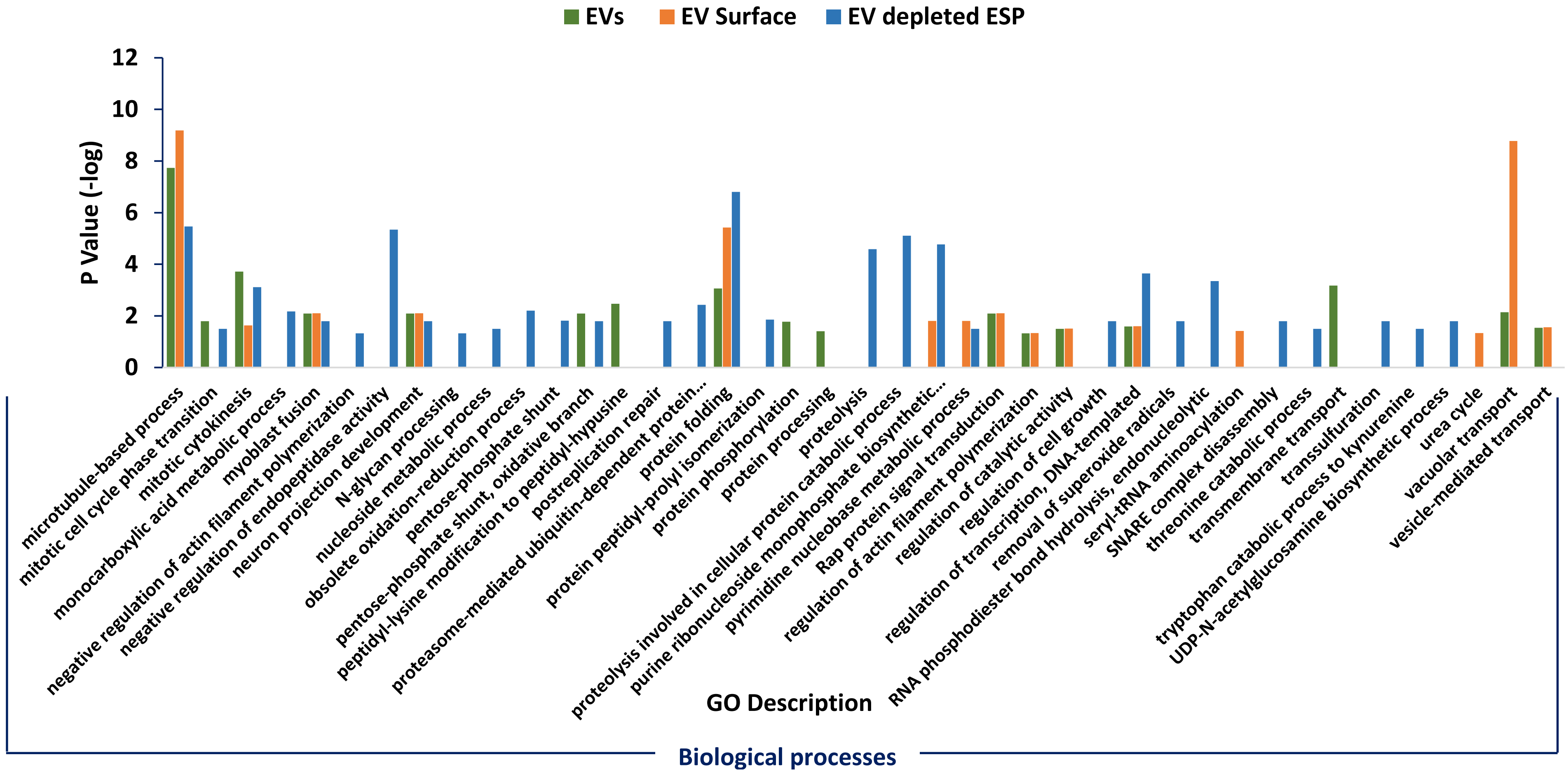

Supplementary Figure S6-continued 2. Gene ontology enrichment analysis using GOATOOLS in python comparing all 3 proteomic datasets from the *A. perfoliata* secretome compared to the whole *A. perfoliata* transcriptome for the Biological process functional category. GO terms were not propagated up the hierarchy (nop) ($p<0.05$ identified significance) and where enriched in all or any of the 3 proteomic datasets were presented using -log transformation of p_uncorrected value and GO description.

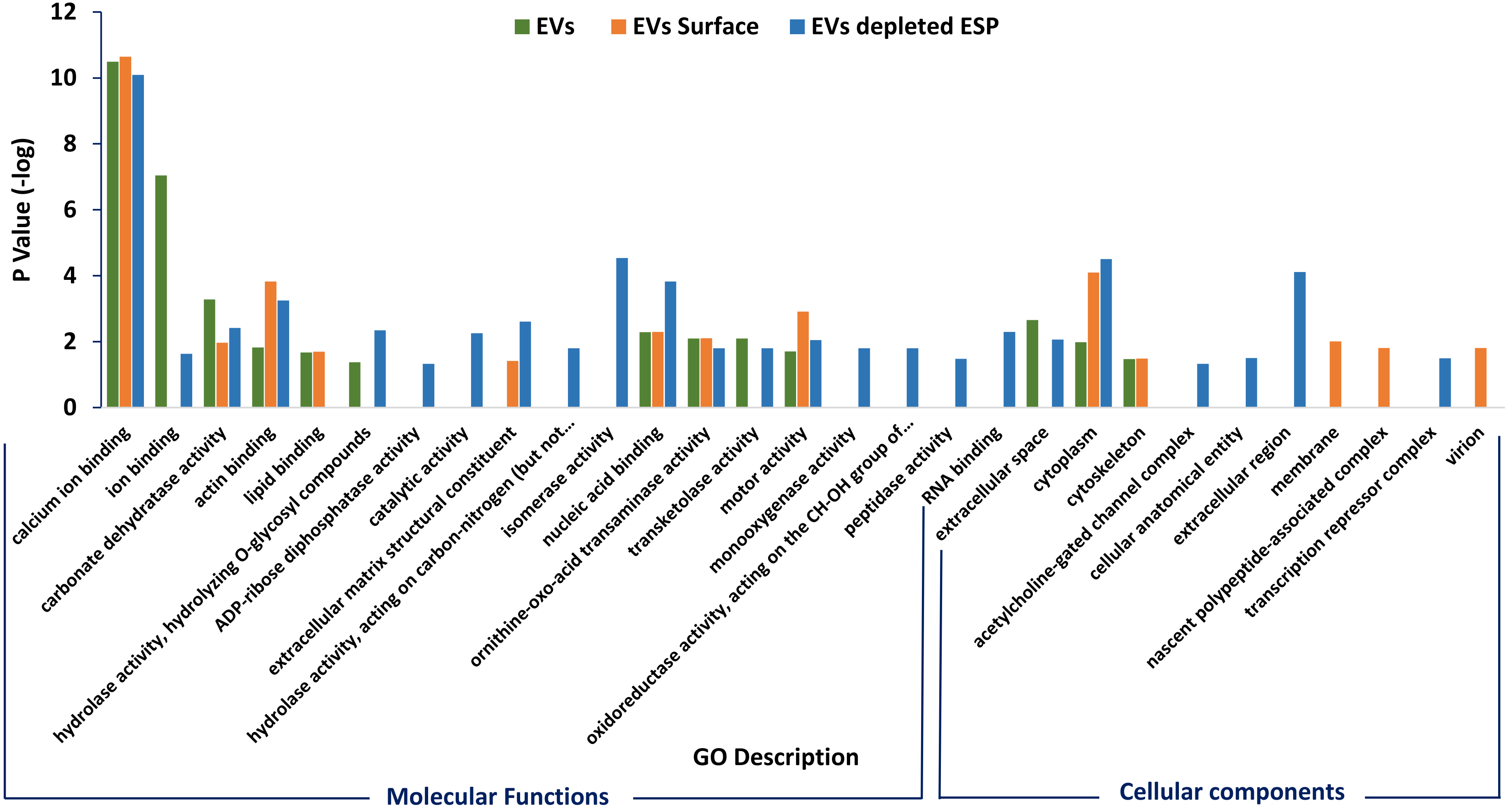

Supplementary Figure S6-continued 3. Gene ontology enrichment analysis using GOATOOLS in python comparing all 3 proteomic datasets from the *A. perfoliata* secretome compared to the whole *A. perfoliata* transcriptome for the Molecular functions and Cellular components functional categories. GO terms were not propagated up the hierarchy (nop) ($p<0.05$ identified significance) and where enriched in all or any of the 3 proteomic datasets were presented using -log transformation of p_uncorrected value and GO description.
